# Functional Analysis of the Coronary Heart Disease Risk Locus on Chromosome 21q22

**DOI:** 10.1155/2017/1096916

**Published:** 2017-03-28

**Authors:** Katherine E. Beaney, Andrew J. P. Smith, Lasse Folkersen, Jutta Palmen, S. Goya Wannamethee, Barbara J. Jefferis, Peter Whincup, Tom R. Gaunt, Juan P. Casas, Yoav Ben-Shlomo, Jacqueline F. Price, Meena Kumari, Andrew Wong, Ken Ong, Rebecca Hardy, Diana Kuh, Nicholas Wareham, Mika Kivimaki, Per Eriksson, Steve E. Humphries, UCLEB Consortium

**Affiliations:** ^1^Centre for Cardiovascular Genetics, British Heart Foundation Laboratories, Institute of Cardiovascular Science, University College London, University Street, London, UK; ^2^Cardiovascular Medicine Unit, Center for Molecular Medicine and Department of Medicine, Karolinska University Hospital Solna, Karolinska Institutet, Stockholm, Sweden; ^3^Center for Biological Sequence Analysis, Technical University of Denmark, Copenhagen, Denmark; ^4^Department of Primary Care & Population Health, UCL Institute of Epidemiology & Health Care, University College London, London, UK; ^5^Population Health Research Institute, St George's University of London, Cranmer Terrace, London, UK; ^6^MRC Integrative Epidemiology Unit, School of Social and Community Medicine, University of Bristol, Bristol, UK; ^7^Farr Institute of Health Informatics Research, University College London, London, UK; ^8^School of Social and Community Medicine, University of Bristol, Bristol, UK; ^9^Centre for Population Health Sciences, University of Edinburgh, Edinburgh, UK; ^10^Department of Epidemiology & Public Health, UCL Institute of Epidemiology & Health Care, University College London, UK; ^11^Institute for Social and Economic Research, University of Essex, Colchester, UK; ^12^MRC Unit for Lifelong Health and Ageing, London, UK; ^13^MRC Epidemiology Unit, Institute of Metabolic Science, Addenbrooke's Hospital, Cambridge, UK; ^14^Institute of Cardiovascular Science, University College London, London, UK

## Abstract

*Background*. The coronary heart disease (CHD) risk locus on 21q22 (lead SNP rs9982601) lies within a “gene desert.” The aim of this study was to assess if this locus is associated with CHD risk factors and to identify the functional variant(s) and gene(s) involved.* Methods*. A phenome scan was performed with UCLEB Consortium data. Allele-specific protein binding was studied using electrophoretic mobility shift assays. Dual-reporter luciferase assays were used to assess the impact of genetic variation on expression. Expression quantitative trait analysis was performed with Advanced Study of Aortic Pathology (ASAP) and Genotype-Tissue Expression (GTEx) consortium data.* Results*. A suggestive association between QT interval and the locus was observed (rs9982601  *p* = 0.04). One variant at the locus, rs28451064, showed allele-specific protein binding and its minor allele showed 12% higher luciferase expression (*p* = 4.82 × 10^−3^) compared to the common allele. The minor allele of rs9982601 was associated with higher expression of the closest upstream genes (*SLC5A3* 1.30-fold increase *p* = 3.98 × 10^−5^;* MRPS6* 1.15-fold increase *p* = 9.60 × 10^−4^) in aortic intima media in ASAP. Both rs9982601 and rs28451064 showed a suggestive association with* MRPS6* expression in relevant tissues in the GTEx data.* Conclusions*. A candidate functional variant, rs28451064, was identified. Future work should focus on identifying the pathway(s) involved.

## 1. Introduction

The coronary heart disease (CHD) risk locus on chromosome 21q22 (lead SNP, rs9982601) is typical of many variants associated with common disease in genome-wide association studies (GWAS) in that the locus falls in an intergenic region and is not associated with any major CHD risk factors [1]. As such, there is no obvious mechanism through which it is affecting CHD risk. Neither the lead SNP at the 21q22 risk locus nor any SNPs in strong linkage disequilibrium (LD) with it result in changes to the protein coding sequence or splice sites in any of the nearby genes. Therefore, the locus does not influence CHD risk by altering protein composition or structure. Rather, it is likely that the locus elicits its effect through involvement in the regulation of gene expression [2]. A feature of the GWAS methodology is that a prior hypothesis of which loci are influencing a trait or disease is not required. Therefore, it has the potential to identify risk variants and ultimately molecular pathways which otherwise would remain obscure. However, to fully take advantage of this, functional analysis of GWAS loci is required to determine the mechanism(s) involved.

The closest downstream gene to the risk locus is the potassium channel subunit encoding* KCNE2. *Mutations in this protein are known to cause long-QT syndrome [3], which is associated with arrhythmias and sudden cardiac death. Furthermore, even within the normal range, longer QT interval has been associated with increased risk of CHD mortality [4], although it is unclear whether the association is a causal relationship. Upstream of the risk locus, the closest genes are* SLC5A3* and* MRSP6*. These genes share an exon which is in the open reading frame for* MRPS6* but not* SLC5A3* [5].* SLC5A3* (solute carrier family 5-inositol transporter) encodes a sodium myoinositol transporter which is involved in the response to hyperosmotic stress [6].* MRPS6* (mitochondrial ribosomal protein 6) encodes a subunit of the mitochondrial ribosome [7]. The lead SNP at this locus, rs9982601, has been found to be associated with expression of* MRPS6* in blood, with the risk allele showing increased expression [8], indicating that risk locus might be involved in regulating the expression of this gene. However, none of these genes point to a plausible pathway to account for the association with CHD and thus genomic location does not suggest any obvious mechanism through which this CHD risk locus is acting.

As part of our ongoing functional analysis of GWAS-identified CHD risk loci we sought to investigate the mechanism through which the 21q22 risk locus influences CHD risk. The aims of this study were firstly to assess whether the risk locus was associated with any traits that may give insight into the mechanism through which the locus affects risk by performing a phenome scan using data from the University College, London School of Hygiene and Tropical Medicine, Edinburgh and Bristol (UCLEB) Consortium of large-scale cohort studies. Secondly, we sought to identify candidate functional SNP(s) using in vitro assays and thirdly to assess relationship between the risk locus and gene expression by performing expression quantitative trait loci (eQTL) analysis with data from the Advanced Study of Aortic Pathology (ASAP) and the Genotype-Tissue Expression (GTEx) consortium.

## 2. Methods

### 2.1. Phenome Scan

The University College, London School of Hygiene and Tropical Medicine, Edinburgh and Bristol (UCLEB) Consortium comprises 12 prospective studies and has been described in detail elsewhere [9]. Approximately 21,000 participants included in the UCLEB studies were genotyped using the Metabochip [10]. This platform has approximately 200,000 SNPs, designed to cover regions associated with cardiometabolic disease. Traits available included a number of physiological phenotypes, lipid measures, and inflammatory markers. Informed consent was obtained for all subjects included in UCLEB research. Written approval from individual Research Ethics Committees to use anonymised individual level data has been obtained by each participating study.

### 2.2. Bioinformatics

HaploReg v2 [11] was used to characterise the lead SNP at the risk locus and those in strong LD with it. LD data came from the 1000 Genomes Project phase 1. The annotations displayed include chromatin state assignments from the ENCODE project [12] and the Roadmap Epigenomics Consortium [13] as well as DNA hypersensitivity site and protein binding annotations from the ENCODE project. Predicted changes in transcription factor binding were assessed using the Genomatix Software Suite (Genomatix Software GmbH, Munich, Germany).

### 2.3. Electrophoretic Mobility Shift Assay (EMSA)

Hepatocellular carcinoma cell lines (Huh-7 and HepG2) were obtained from the European Collection of Cell Cultures. The cells were cultured and nuclear proteins extracted as described in [14]. Probes with 25 bases flanking the SNP of interest were designed (two per SNP, one for each allele), biotinylated, and annealed to form double stranded oligonucleotides. Probe sequences are given in Supplementary Table  1, in Supplementary Material available online at https://doi.org/10.1155/2017/1096916. The probes were incubated with nuclear extract (with or without competitor) at 25°C for 50 minutes. Following this the reactions were run on a 6% polyacrylamide gel for 210 minutes at 120 V and thereafter transferred on to Hybond-N+ membrane using Southern transfer [15]. DNA-protein complexes were cross-linked to the membrane and visualised and using the Thermo Scientific Lightshift Chemiluminescent Nucleic Acid Detection Module (according to the manufacturer's instructions, Thermo Fisher Scientific, Waltham, MA, USA).

### 2.4. Dual-Reporter Luciferase Assay

The region surrounding the putative functional SNP was cloned into the pGL3 promoter vector (Promega, Madison, WI, USA) at the enhancer region using the SalI/BamHI restriction sites. Huh-7 cells were seeded 1–5 × 10^−4^ cells/well and grown to near confluence. The cells were then transfected using Lipofectamine (Invitrogen, Life Technologies, Carlsbad, CA, USA) according to the manufacturer's instructions and left for 48 hours. Reporter plasmid DNA and cotransfectant DNA (pRL-TK) were added to each well in a ratio of 200 : 1. Luciferase activity was determined by measuring the luminescence of the cell lysate using the TR717 Microplate Luminometer and the Dual Luciferase assay kit (Promega, Madison, WI, USA) according to the manufacturer's instructions. The ratio of firefly luciferase readings:* Renilla* luciferase readings was then compared between the different constructs using paired *t*-tests. Four runs of eleven or twelve replicates were performed.

### 2.5. eQTL Analysis

#### 2.5.1. ASAP

Patients undergoing aortic value surgery at the Karolinska University Hospital, Stockholm, Sweden, were recruited (*n* = 213) into ASAP [16]. Tissue biopsies were taken from the mammary artery, aortic adventitia, aortic intima media, heart, and liver. Messenger RNA was extracted and measured using the Affymetrix Gene Chip Human Exon 1.0 ST Expression Array (Santa Clara, CA, USA). Genotyping was performed using the Illumina Human 610 W Quad Beadarrays (San Diego, CA, USA). All participants gave informed consent and the study had approval from the ethical committee of the Karolinska Institute. The ASAP data was used to assess the relationship between the risk locus and the closest upstream and downstream genes—*SLC5A3*,* MRPS6,* and* KCNE2*—in the five available tissues.

#### 2.5.2. GTEx

The GTEx consortium seeks to provide a resource to study the relationship between genetic variation and gene expression by collecting multiple samples from densely genotypes donors [17]. The data is publically available on the GTEx browser (http://gtexportal.org/home/). The GTEx data was used to assess the relationships between SNPs at the risk locus with* MRPS6* and* KCNE2* expression in the aortic artery, coronary artery, tibial artery, atrial appendage, left ventricle liver, and whole blood.

## 3. Results

### 3.1. Association of the 21q22 CHD Risk Locus with CHD Risk Factors

A phenome scan of the UCLEB Consortium data was performed to determine if SNPs at the 21q22 risk locus were associated with risk factors for CHD. Four SNPs at the 21q22 locus were analysed, the lead SNP rs9982601 and three SNPs in moderate LD with it, rs8131284 (*r*^2^ = 0.78), rs7278204 (*r*^2^ = 0.76), and rs973754 (*r*^2^ = 0.75). Over one hundred traits were tested (these are listed in the Appendix) but none met the Bonferroni-adjusted significance threshold (*p* = 4.72 × 10^−4^, based on an unadjusted significance threshold of *p* = 0.05). Only one trait, QT interval, showed a suggestive association (*p* < 0.05) with the effect in the same direction for all four SNPs (Table 1). The minor CHD risk allele was nominally associated with longer QT interval. This putative association is of interest due to the close proximity of the potassium ion channel gene* KCNE2* to the risk locus.

### 3.2. Identification of a Putative Functional SNP

#### 3.2.1. Bioinformatics Analysis of the CHD Risk Locus 21q22

Five SNPs in strong LD (*r*^2^ > 0.8) with the lead SNP rs9982601 were identified using HaploReg v2 [11]. Only one SNP (rs28451064) resided within an enhancer region (in the liver cell line HepG2). This SNP was also found to be positioned within a site bound by multiple transcription factors including specificity protein 1 (SP1) and forkhead box A2 (FOXA2), also in HepG2 cells.

#### 3.2.2. Assessment of Allele-Specific Binding

**Figure 1:**
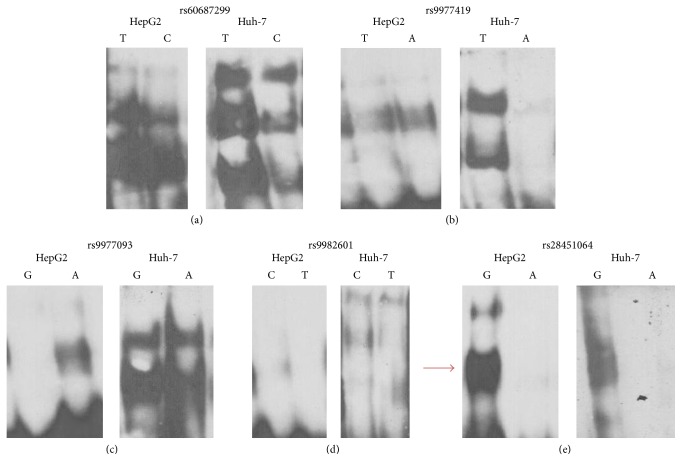
Replication of EMSAs with (a) rs60687299, (b) rs977419, (c) rs977093, (d) rs9982601, and (e) rs28451064 probes using HepG2 and Huh-7 nuclear extract. Binding by both alleles was compared for all five SNPs. Only in (e) (rs28451064) is there strong binding for one allele but complete absence of binding for the other in both cell lines.

EMSAs were performed for the lead SNP and the five in strong LD with it, to assess if any of the SNPs showed allele-specific binding. The assays were carried out with nuclear extracts from two hepatocyte carcinoma cell lines, HepG2 and Huh-7, as the only enhancer chromatin marks found for any of the SNPs at this locus were in HepG2 cells. Only rs28451064 showed consistent allele-specific binding (Figure 1). Therefore, the result of the EMSAs together with the bioinformatics analysis identifies rs28451064 as a strong candidate to be the functional SNP.

#### 3.2.3. Impact of rs28451064 on Transcription Factor Binding

To assess whether transcription factor binding may be affected by the presence of different alleles at rs28451064, the Genomatix Software Suite (Genomatix Software GmbH, Munich, Germany) was used. Presence of the minor “A” allele rather than the major “G” allele was predicted to abolish a vitamin D receptor-retinoid X receptor (VDR-RXR) heterodimer binding site and a homeodomain protein H6 family member 3 (HMX3) binding site. The software also predicted the creation of a forkhead-related transcription factor 4 (FREAC4) binding site in the presence of the (minor) A allele.

**Figure 2:**
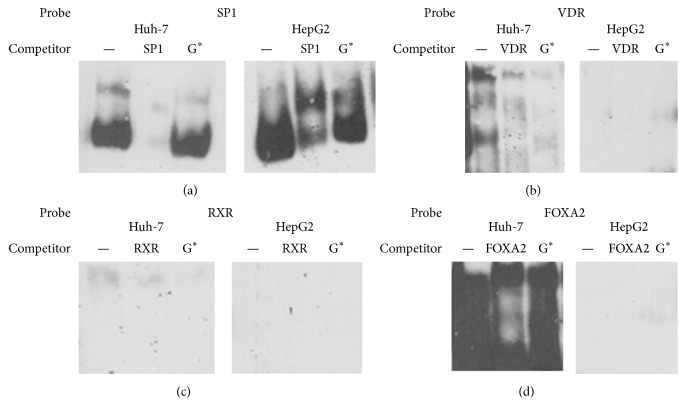
Competitor EMSA results from assays performed with (a) SP1 probes, (b) VDR probes, (c) RXR probes, and (d) FOXA2 probes and HepG2 and Huh-7 nuclear extracts. ^*∗*^G allele of rs28451064.

To investigate whether VDR or RXR were binding to the rs28451064 probes in vitro, competitor EMSAs were performed with both Huh-7 and HepG2 nuclear extracts. SP1 and FOXA2 were also investigated as these proteins had been found to bind at this locus in the HepG2 cell line in the ENCODE project (Figure 2). SP1 binding to its consensus sequence was not competed out by the addition of the rs28451064-G probe and the RXR consensus probe did not bind proteins in either extract, demonstrating that these were unlikely to be the bound factor. There were no consistent binding results with FOXA2 and VDR, and thus no firm conclusions on their binding to rs28451064 in vitro could be drawn.

#### 3.2.4. Impact of rs28451064 on Reporter Gene Expression

**Figure 3:**
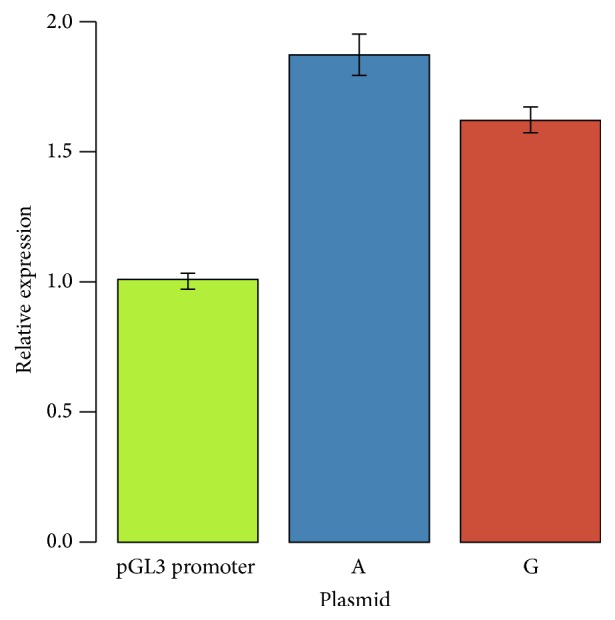
Relative expression of a vector containing the rs284510654 A allele and rs28451064 G allele normalised to the pGL3 promoter expression. Relative expression was compared using paired *t*-tests. Both plasmids containing the sequence surrounding rs28451064 showed higher expression (A allele 87% higher *p* = 1.90 × 10^−15^, G allele 62% higher *p* = 9.74 × 10^−15^). The A allele was found to have 12% higher expression compared to the G allele (*p* = 4.82 × 10^−3^).

The impact of rs28451064 on gene expression was assessed using a luciferase dual-reporter assay. To do this, a fragment of 300 bp containing the SNP was cloned into the enhancer site of the pGL3 promoter vector and the plasmids transfected into Huh-7 cells.  Figure 3 shows the relative expression of the reporter gene containing the rs28451064 insert compared to the pGL3 promoter plasmid. Both rs28451064 plasmids showed higher expression (A allele 87% higher *p* = 1.90 × 10^−15^, G allele 62% higher *p* = 9.74 × 10^−15^) than the pGL3 promoter plasmid, suggesting that this region acts as an enhancer. Moreover, in agreement with the eQTL data presented below, the minor A (risk) allele was found to have 12% higher expression compared to the G allele (*p* = 4.82 × 10^−3^).

### 3.3. eQTL Analysis

**Figure 4:**
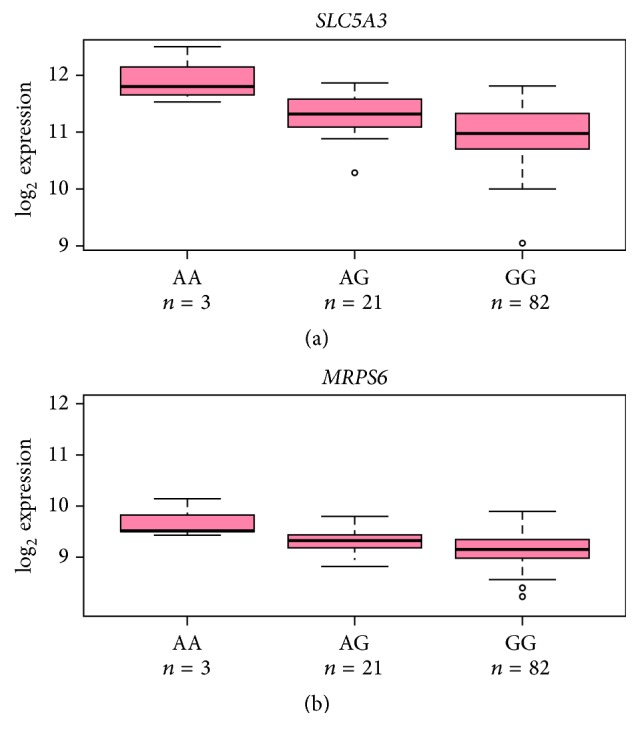
Expression of (a)* SLC5A3* and (b)* MRPS6* in aortic intima media presented by rs9982601 genotype in the ASAP study. The minor allele of rs9982601 was associated with higher expression of both* SLC5A3* (1.30-fold (95% CIs 1.16–1.47) per A allele *p* = 3.98 × 10^−5^) and* MRPS6* (1.15-fold (95% CIs 1.06–1.25) per A allele *p* = 9.60 × 10^−4^).

The relationship between the lead SNP at the risk locus and expression of its three closest genes* MRPS6*,* SLC5A3,* and* KCNE2* was examined using data from the ASAP study [16]. Expression levels in five tissues (liver, mammary artery, aortic adventitia, aortic intima media, and heart) and rs9982601 genotype data was available for 106 ASAP participants. The minor CHD risk allele was associated with higher expression of the mRNA transcript in aortic intima media for both* SLC5A3* and* MRPS6* (*SLC5A3* 1.30-fold (95% CIs 1.16–1.47) per A allele *p* = 3.98 × 10^−5^;* MRPS6* 1.15-fold (95% CIs 1.06–1.25) per A allele *p* = 9.60 × 10^−4^, Figure 4). However, no association was observed for* KCNE2*. Similar results were observed for rs28451064 (*SLC5A3* 1.40-fold increase per minor allele, *p* = 1.08 × 10^−6^;* MRPS6* 1.20-fold increase per minor allele, *p* = 1.47 × 10^−5^).

The relationship between the 21q22 CHD risk locus and gene expression was further studied using publically available data from the GTEx project (http://www.gtexportal.org/) [17]. No genes met the significance threshold for a single tissue eQTL with either the lead SNP rs9982601 or the putative functional SNP rs28451064. The search was then narrowed to consider the relationship between the risk locus and the three genes located most closely to it,* KCNE2*,* MRPS6,* and* SLC5A3*, in seven relevant tissues (Table 2). This gives a Bonferroni-adjusted* p* value of 4 × 10^−3^ (based on an unadjusted significance threshold of *p* = 0.05). In agreement with the ASAP results, the minor allele of rs9982601 was found to be associated with higher expression of* MRPS6* in the aortic (effect of minor allele relative to common allele = 0.15, *p* = 3.5 × 10^−3^) and tibial arteries (effect of minor allele relative to common allele = 0.15, *p* = 1.8 × 10^−4^), although not in the coronary artery. There was a suggestive association between the minor allele and lower expression of* MRPS6* in whole blood (effect of minor allele relative to common allele = −0.09, *p* = 0.04). There were suggestive associations between the minor allele of rs9982601 and higher expression of* KCEN2* in aortic and tibial artery tissue (effect of minor allele relative to common allele = 0.13, *p* = 0.06, and effect of minor allele relative to common allele = 0.15, *p* = 0.02, resp.). Expression data for* SLC5A3* was not available. Results for rs28451064 were very similar (data not shown).

## 4. Discussion

The locus on chromosome 21q22 has been consistently associated with CHD, with odds ratio of 1.13 per minor allele in the CARDIoGRAMplusC4D meta-analysis [1], which is relatively high for a GWAS-identified risk variant. However, like the majority of the confirmed GWAS loci for CHD, there is no obvious functional mechanism through which the locus influences CHD risk [1]. In this study, rs28451064 was identified as a putative functional SNP at the locus. The minor CHD risk allele was found to show less protein binding and be associated with higher gene expression in vitro. This allele was also found to be associated with higher expression of the two closest upstream genes (*MRPS6* and* SLC5A3*) in a number of tissues. In agreement with previous studies, no significant association between the lead SNP, rs9982601, and risk factors for CHD was observed. A suggestive association between the risk locus and QT interval was observed, indicating that it may be affecting CHD risk through regulating the expression of the potassium channel subunit gene* KCNE2*, the closest downstream gene to the risk locus. However, while a suggestive association between rs9982601 and* KCNE2* expression in the aortic and tibial arteries was observed in the GTEx data, the evidence for the risk locus being involved in the regulation of* MRPS6* and* SLC5A3* was more consistent. These data raise the possibility that the risk locus is acting on more than one pathway influencing the development of CHD.

While a putative functional SNP was identified, the transcription factors involved remain to be identified. The minor (risk) allele of rs28451064 was predicted to abolish a binding site for the VDR-RXR heterodimer transcription factor complex. A large-scale analysis performed in lymphoblastoid cells found that expression of both* MRPS6* and* SLC5A3* increased in response to treatment with calcitriol (a bioactive form of vitamin D) [18]. This suggests that the VDR pathway is involved in expression of these two genes, although if so, it is not a simple relationship given that higher expression of both genes is associated with the minor (risk) allele which is also predicted to abolish the VDR binding site. The competitor EMSAs performed herein investigated which transcription factors were binding the rs28451064-G probe; however the results were inconsistent and VDR binding to the probe could neither be confirmed nor be refuted.

How increased expression of any of the nearby genes (*MRPS6*,* SLC53,* and* KCNE2*) might affect CHD risk is unclear. As a constituent part of the mitochondrial ribosome, the gene product of* MRPS6* plays a key role in the synthesis of the thirteen proteins encoded in mitochondrial DNA, all of which are involved in oxidative phosphorylation [19]. An important by-product of oxidative phosphorylation is the generation of reactive oxygen species (ROS) [20]. Overproduction of ROS by dysfunctional mitochondria has been associated with multiple proatherogenic consequences including the activation of inflammatory pathways and endothelial dysfunction, but whether this is a causal relationship remains to be determined [21]. If so, it may be that increased expression of* MRPS6* caused by presence of the risk allele disrupts the translation of the genes encoded by the mitochondrial DNA, increasing the risk of mitochondrial dysfunction and ultimately oxidative stress.

The sequence of* SLC5A3* lies completely within that of* MRPS6*. The protein encoded by* SLC5A3* is a sodium-myoinositol cotransporter (SLC5A3). This transporter plays an important role in the maintenance of cell volume in response to hyperosmotic stress. As osmolarity of the extracellular fluid increases, nonselective cation channels are activated causing sodium ions to enter the cell, disrupting cellular ion homeostasis. In response, solute carrier family proteins (including SLC5A3) are activated causing increased transport of small organic molecules such as myoinositol, referred to as “compatible osmolytes” to replace inorganic ions [22]. Recent evidence has suggested that SLC5A3 is also involved in the response to hypotonic stress but this is much less well understood [23]. Work in mouse models has found that SLC5A3 is involved in the development of the peripheral nervous system and respiratory gas exchange [24, 25]. From current knowledge there is no obvious mechanism to link SLC5A3 with the pathogenesis of CHD. However, as there may be a number of pathways which contribute to atherosclerosis yet to be elucidated (indicated by the number of GWAS hits with unknown mechanisms), the involvement of SLC5A3 cannot be discounted. Alternatively, the association between the risk locus and expression of this gene may simply be a consequence of its sharing an exon with* MRPS6* [5].

The relationship between* KCNE2* and CHD appears to be complex. The ion channel subunit encoded by* KCNE2* has a long established relationship with QT interval (and thus with the electrical activity of the ventricles). How this may relate to CHD risk is yet to be determined. Recently, deletion of the gene was found to promote spontaneous atherosclerotic lesions in mice [26]. These mice also had raised LDL-cholesterol and impaired glucose tolerance, both proatherogenic characteristics [27]. While results from mice are not directly translatable to humans, this does provide preliminary evidence of a causal relationship between* KCNE2* and CHD. However, only weak evidence for a relationship between the 21q22 risk locus and the gene was observed in this study.

This work has a number of limitations. Functional molecular assays were performed in hepatocyte carcinoma cell lines and these may not be the most appropriate cellular model. However, the putative functional SNP, rs28451064, was found to have enhancer chromatin marks in HepG2 (hepatocyte) cells and lie in both a DNAse I hypersensitivity site and transcription binding sites. This indicates that the SNP lies in open chromatin in this cell line and thus may be influencing gene expression. Furthermore, since the mechanism through which this locus impacts upon risk remains obscure, it is not clear which cell type would serve as the most appropriate model. The luciferase assays were performed using the pGL3 promoter vector which contains a general SV40 bacterial promoter. It would have been preferable to use the promoter of either* MRPS6* or* SLC5A3*. However, the* MRPS6* promoter is not well characterised and attempts to clone the* SLC5A3* promoter sequence into the pGL3 basic plasmid were unsuccessful. A further limitation is that the in vitro assays performed herein are unable to take account of chromatin state or to assess long distance interactions. However, chromatin capture techniques are now in mainstream use which can overcome this issue. The methods can be used to investigate whether two loci interact (chromatin conformation capture, 3C [28]) or to identify interaction partners for a particular locus circular chromosome confirmation capture [29]. Future work on this locus should focus on this.

In conclusion, functional analysis of the 21q22 CHD risk locus was performed, identifying a putative functional SNP, rs28451064. However, the affected gene(s) and transcription factor(s) remain obscure. Future work should focus on identifying the pathway(s) through which this locus influences CHD risk, specifically the transcription factors and genomic loci involved.

## Supplementary Material

Supplementary Table 1: Probe sequences for SNPs at the 21q22 CHD risk locus.

## Figures and Tables

**Table 1:** The association between four SNPs at the CHD risk locus on chromosome 21q22 and mean QT interval in UCLEB.

SNP					Beta coefficient	*p* value
rs9982601	Genotype	CC	TC	TT	1.83 (0.90)	0.04
	*n* (frequency)	5329 (0.75)	1643 (0.23)	130 (0.02)		
	Mean QT interval (ms)	402.8 (37.35)	404.5 (39.28)	409.1 (41.08)		

rs8131284	Genotype	TT	CT	CC	2.14 (0.89)	0.02
	*n* (frequency)	5293 (0.75)	1670 (0.24)	142 (0.02)		
	Mean QT interval (ms)	402.7 (37.41)	404.9 (39.20)	408.1 (38.97)		

rs7278204	Genotype	AA	GA	GG	2.07 (0.89)	0.02
	*n* (frequency)	5290 (0.74)	1673 (0.24)	141 (0.02)		
	Mean QT interval (ms)	402.7 (37.73)	404.9 (38.19)	407.9 (39.26)		

rs973754	Genotype	AA	GA	GG	1.85 (0.89)	0.02
	*n* (frequency)	5300 (0.75)	1663 (0.23)	142 (0.02)		
	Mean QT interval (ms)	402.7 (37.75)	404.6 (38.18)	408.2 (38.84)		

Mean QT interval is shown by genotype as well as the beta coefficient (±standard deviation) for the minor allele at each SNP.

**Table 2:** Relationship between rs9982601 and expression of selected genes in seven tissues from GTEx (http://www.gtexportal.org/home/).

Gene	Tissue	*n*	Effect size (se)	*p* value
*MRPS6*	Aortic artery	197	0.15 (0.05)	3.5 × 10^−3^
*MRPS6*	Coronary artery	118	0.14 (0.11)	0.22
*MRPS6*	Tibial artery	285	0.15 (0.05)	1.8 × 10^−3^
*MRPS6*	Atrial appendage	159	−0.02 (0.09)	0.88
*MRPS6*	Left ventricle (heart)	190	−0.16 (0.10)	0.09
*MRPS6*	Liver	97	−0.19 (0.14)	0.17
*MRPS6*	Whole blood	338	−0.09 (0.04)	0.04
*KCNE2*	Aortic artery	197	0.13 (0.07)	0.06
*KCNE2*	Coronary artery	118	0.09 (0.11)	0.43
*KCNE2*	Tibial artery	285	0.15 (0.06)	0.02
*KCNE2*	Atrial appendage	159	0.13 (0.16)	0.41
*KCNE2*	Left ventricle (heart)	190	−0.06 (0.14)	0.71
*KCNE2*	Liver	97	0.05 (0.15)	0.31
*KCNE2*	Whole blood	338	8.40 × 10^−3^ (0.15)	0.92

Effect sizes refer to the effect of the minor allele on expression relative to the common allele. se = standard error.

## Appendix

The traits included in the phenome scan were as follows: albumin, alcohol use, alkaline phosphate, alanine transaminase, apoa1, apob, activated partial thromboplastin time, aspartate aminotransferase, basophil count, bilirubin, body mass index, use of blood pressure lowering medication, electrocardiogram cornell product, creatinine, C-reactive protein, diastolic blood pressure, D-dimer, estimated glomerular filtration rate, eosinophil count, factor VII, factor VIII, ferritin, forced expiatory volume in 1 second, fibrinogen, forced vital capacity, gamma-glutamyl transpeptidase, glucose, use of glucose lowering drugs, haemoglobin, glycated haemoglobin, haematocrit, homocysteine, high density lipoprotein cholesterol, height, insulin-like growth factor-1, interleukin-6, insulin, low density lipoprotein cholesterol, log* *(alkaline phosphate cholesterol), log* *(alanine transaminase), log* *(aspartate aminotransferase), log* *(basophil), log* *(bilirubin), log* *(electrocardiogram cornell product), log* *(C-reactive protein), log* *(D-dimer), log* *(eosinophil count), log* *(factor VII), log* *(ferritin), log* *(fibrinogen), log* *(gamma-glutamyl transpeptidase), log* *(glucose), log* *(homocysteine), log* *(interleukin-6), log* *(insulin), log* *(lipoprotein (a)), log* *(lymphocyte count), log* *(monocyte count), log* *(electrocardiogram QRS voltage product), log* *(electrocardiogram QRS voltage sum), log* *(serum magnesium), log* *(serum phosphate), log* *(serum urea), log* *(triglycerides), log* *(tumor necrosis factor-alpha), log* *(uric acid), log* *(von Willebrand factor), log* *(white cell count), lipoprotein (a), lymphocyte count, mean cell haemoglobin, mean cell volume, monocyte count, mean platelet volume, neutrophil count, body fat percentage, peak expiratory flow, platelet count, PR interval, electrocardiogram QRS voltage product, electrocardiogram QRS voltage sum, electrocardiogram QT interval corrected, electrocardiogram QT interval, red blood cell count, systolic blood pressure, serum calcium concentration, serum magnesium concentration, smoking, electrocardiogram Sokolow-Lyon, serum phosphate concentration, serum potassium concentration, total serum protein concentration, serum sodium concentration, serum urea concentration, total cholesterol, triglycerides, tumor necrosis factor-alpha, uric acid, plasma viscosity, von Willebrand factor, white cell count, waist circumference, waist-to-hip ratio, and weight.

## Additional Points

*DOI.* Data used in this publication are available to bona fide researchers upon request to the NSHD Data Sharing Committee via a standard application procedure. Further details can be found at http://www.nshd.mrc.ac.uk/data, doi: 10.5522/NSHD/Q101. The UCLEB Consortium is composed of the following individuals: Tina Shah, Jorgen Engmann, Chris Finan, Amand Floriaan Schmidt, and Aroon D. Hingorani: Institute of Cardiovascular Science, University College London, London, United Kingdom; Caroline Dale, Pimphen Charoen, Antoinette Amuzu, Ghazaleh Fatemifar, Juan P. Casas, and Claudia Langenberg: Farr Institute of Health Informatics, University College London, London, United Kingdom; Jon White and Vincent Plagnol: University College London Genetics Institute, Department of Genetics, Environment and Evolution, London, United Kingdom; Frank Dudbridge: Department of Non-Communicable Disease Epidemiology, London School of Hygiene and Tropical Medicine, London, United Kingdom; Meena Kumari: Institute for Social and Economic Research, University of Essex, Colchester, United Kingdom; Meena Kumari and Mika Kivimaki: Department of Epidemiology & Public Health, UCL Institute of Epidemiology and Health Care, University College London, London, United Kingdom; Stela McLachlan and Jacqueline Price: Centre for Population Health Sciences, University of Edinburgh, Edinburgh, United Kingdom; Christine Power and Elina Hypponen: MRC Centre of Epidemiology for Child Health, Department of Population Health Sciences, UCL Institute of Child Health, University College London, London, United Kingdom; Andrew Wong, Ken Ong, Rebecca Hardy, and Diana Kuh: MRC Unit for Lifelong Health and Ageing, London, United Kingdom; Ken Ong and Nicholas Wareham: MRC Epidemiology Unit, Institute of Metabolic Science, Addenbrooke's Hospital, Cambridge, United Kingdom; Tom R. Gaunt, Debbie A. Lawlor, George Davey Smith, and Fotios Drenos: MRC Integrative Epidemiology Unit, School of Social and Community Medicine, University of Bristol, Bristol, United Kingdom; Fotios Drenos, Jackie Cooper, Philippa J. Talmud, and Steve E. Humphries: Centre for Cardiovascular Genetics, Department of Medicine, British Heart Foundation Laboratories, Rayne Building, Royal Free and University College Medical School, London, United Kingdom; Reecha Sofat: Centre for Clinical Pharmacology, University College London, London, United Kingdom; Yoav Ben-Shlomo: School of Social and Community Medicine, University of Bristol, Bristol, United Kingdom; Peter Whincup: Population Health Research Institute, St George's University of London, London, United Kingdom; Richard Morris: School of Social and Community Medicine, University of Bristol, Bristol, United Kingdom; Barbara Jefferis and Goya Wanamethee: Department of Primary Care and Population Health, UCL Institute of Epidemiology and Health Care, University College London, London, United Kingdom; Claudia Langenberg: MRC Epidemiology Unit, University of Cambridge School of Clinical Medicine, Institute of Metabolic Science, Cambridge Biomedical Campus, Cambridge, United Kingdom.
