# Supplementary material for: Functional Analysis of the Coronary Heart Disease Risk Locus on Chromosome 21q22
Source: Dis Markers. 2017 Mar 28;2017:1096916. doi: 10.1155/2017/1096916 (PMC5387827; doi:10.1155/2017/1096916)
Supplement: Supplementary file 1 — Supplementary Table 1: Probe sequences for SNPs at the 21q22 CHD risk locus. [file 1096916.f1.pdf]

Supplementary Table 1: Probe sequences for SNPs at the 21q22 CHD risk locus

SNP - Allele	Sequence
rs9982601 – C	CACAGGGCTGCTCCATGGCCTTGGA
rs9982601 – C	TCCAAGGCCATGGAGCAGCCCTGTG
rs9982601 – T	CACAGGGCTGCTTCATGGCCTTGGA
rs9982601 – T	TCCAAGGCCATGAAGCAGCCCTGTG
rs28451064 – G	CCAGGCCAAAGTGGACACCAAATAC
rs28451064 – G	GTATTTGGTGTCCACTTTGGCCTGG
rs28451064 – A	CCAGGCCAAAGTAGACACCAAATAC
rs28451064 – A	GTATTTGGTGTCTACTTTGGCCTGG
rs9980618 – C	AGGGTGTCTGCTCCAGCACACCATG
rs9980618 – C	CATGGTGTGCTGGAGCAGACACCCT
rs9980618 – T	AGGGTGTCTGCTTCAGCACACCATG
rs9980618 – T	CATGGTGTGCTGAAGCAGACACCCT
rs60687299 – T	CACTGTATTGAATACTGGAGGCAAC
rs60687299 – T	GTTGCCTCCAGTATTCAATACAGTG
rs60687299 – C	CACTGTATTGAACACTGGAGGCAAC
rs60687299 – C	GTTGCCTCCAGTGTTCAATACAGTG
rs9977419 – T	TGTGATAGTGAGTGAGTTCTTACGA
rs9977419 – T	TCGTAAGAACTCACTCACTATCACA
rs9977419 – A	TGTGATAGTGAGAGAGTTCTTACGA
rs9977419 – A	TCGTAAGAACTCTCTCACTATCACA
rs9977093 – G	CCATGCAGAACTGTGAATCAATTAA
rs9977093 – G	TTAATTGATTCACAGTTCTGCATGG
rs9977093 – A	CCATGCAGAACTATGAATCAATTAA
rs9977093 – A	TTAATTGATTCATAGTTCTGCATGG

The SNP position is underlined. EMSA=electrophoretic mobility shift assay.
